# Surgical outcomes of elderly patients aged more than 80 years with distal radius fracture: comparison of external fixation and locking plate

**DOI:** 10.1186/s12891-020-3101-3

**Published:** 2020-02-10

**Authors:** Yu-Yi Huang, Tung-Yi Lin, Chien-Hao Chen, Ying-Chao Chou, Chun-Yi Su

**Affiliations:** 1 Department of Orthopaedic Surgery, Chang Gung Memorial Hospital, Keelung branch, Bone and Joint Research Center, and Chang Gung University, F7, No 222 Mai-King Road, Keelung, Taiwan; 2 Department of Orthopedic Surgery, Division of Trauma, Bone and Joint Research Center, Chang Gung Memorial Hospital and Chang Gung University, Taoyuan, Taiwan

**Keywords:** Elderly, Distal radius fracture, Volar locking plate, External fixation, Surgical treatment

## Abstract

**Background:**

To compare the outcomes after surgical intervention, including external fixation (EF) with the optional addition of K-pins or open reduction and internal fixation (ORIF) with a volar locking plate (VLP), in patients with distal radius fracture aged > 80 years.

**Methods:**

We reviewed 69 patients with a distal radius fracture aged > 80 years who treated under surgical intervention from 2011 to 2017 retrospectively. Their demographic data and complications were recorded. Preoperative, postoperative, and last follow-up plain films were analyzed. The functional outcomes of wrist range of motion were also evaluated.

**Results:**

41 patients were treated with EF with the optional addition of K-pins, while 28 patients were treated with ORIF with a VLP. The radiological parameters, including ulnar variance and radial inclination, at the last follow-up were significantly more acceptable in the VLP group (*p* = 0.01, *p* = 0.03, respectively). The forearm supination was significantly better in patients treated with VLP (*p* = 0.002). The overall incidence of complications was lower in the VLP group (*p* = 0.003).

**Conclusion:**

VLP provides better radiological outcomes, wrist supination and lower complication rates than EF. Therefore, although EF is still widely used because of its acceptable results and easy application, we recommend VLP as a suitable treatment option for distal radius fracture in the geriatric population aged > 80 years.

## Background

The distal radius is one of the most commonly fractured bones seen in the emergency room, accounting 18% of all fractures in the elderly population [1, 2]. As described in the American Academy of Orthopaedic Surgeons clinical practice guideline on distal radius fracture, they were unable to recommend for or against surgical treatment of distal radius fractures in the elderly [3]. In the emergency room, the primary management of distal radius fractures is nonsurgical treatment via closed reduction with cast immobilization. Some studies suggested that patients > 65 years with distal radius fractures treated conservatively with cast immobilization had satisfactory outcomes [4–7]. However, the rate of secondary displacement was significantly increased with age [8] and up to 50% of cases who treated with closed reduction had the incidence of malunion or radiological osteoarthritis at the final follow up [9, 10]. Therefore, surgical treatment may be considered in elderly patients with distal radius fracture.

The surgical treatment options for distal radius fracture in adults include external fixation (EF) and open reduction and internal fixation (ORIF) with a plate. EF is traditionally used in the treatment of distal radius fracture because of its easy application and minimal surgical exposure. ORIF with a plate provides strong fixation for patients with comminuted fractures. By 2050, the life expectancy of the global population is estimated to increase to 81.1 and 86.6 years among men and women, respectively [11]. More active lifestyle and functional demands have mandated increased attention to the fracture management, even in elderly patients. However, there is no literature focus on whether EF or ORIF is the optional treatment for distal radius fractures in patients aged > 80 years. So our study aimed to compare the surgical outcomes of EF and ORIF with a volar locking plate (VLP) in patients aged > 80 years.

## Methods

This study was a retrospective cohort study which reviewed patients between January 2011 and July 2017 after receiving institutional review board approval. The inclusion criteria were patients aged > 80 years with a dorsally displaced, distal radius fracture treated with EF or ORIF with a VLP in our institution. We excluded patients with open fractures and/or concomitant injuries. Patients who needed extra procedures such as bone grafting or bone substitutes for fractures were also excluded. There were 74 patients assessed for eligibility in this study. 2 patients who were lost to follow up before 12 months postoperatively and 3 patients expired during the follow-up duration were excluded. There were total 69 patients enrolled in this study for the final analysis. Their demographic data, including age, sex, mechanism of injury, length of hospital stay, chronic diseases, and personal history were recorded.

### Preoperative

Preoperative plain anteroposterior- and lateral-view radiographs were evaluated. The important parameters of volar tilt, radial inclination, radial height, and ulnar variance were also recorded. All fractures were classified according to the AO/Orthopaedic Trauma Association classification system from the radiographs [12] by two independent reviewers.

### Perioperative

All surgeons had experience of using EF or VLP to treat distal radius fractures. The principle to determine the surgery of EF or VLP was based on the surgeon’s preference. An acceptable reduction was defined as ≤10° dorsal angulation and ≤ 2 mm radial shortening intraoperatively.

#### External fixation

After closed reduction of the distal radius was performed using traction force, two percutaneous Kirschner-pins were introduced dorsolaterally to maintain the fracture reduction. Two distal external pins were attached in the 2nd proximal metacarpal bone. Two proximal external pins were applied via two small dorsolateral incisions made proximal to the extensor pollicis longus muscle and retracted the extensor carpi radialis longus and brevis tendons. Then, the external fixator frame was applied under fluoroscopic monitoring. Active finger range of motion was started immediately after operation. Two weeks after operation, the dressing and suture were removed. The external fixator and K-pins were extracted 6–7 weeks postoperatively in the clinic then wrist active and passive exercises were started.

#### VLP fixation

The surgical exposure of the fracture was based on Henry’s approach and the pronator quadratus muscle was incised on its radial border. The fracture was exposed and reduced. The VLP and screws were used for fracture stabilization by fluoroscopic monitoring; if possible, the pronator quadratus muscle was repaired. In this group, the volar short arm splint was placed for immobilization after operation. Active finger range of motion was started after the day of operation. Two weeks after operation, the dressing and suture were removed and active and passive exercises of the wrist were performed. Besides, the removable splint was used for an additional 2 weeks.

### Postoperative follow-up

**Fig. 1:**
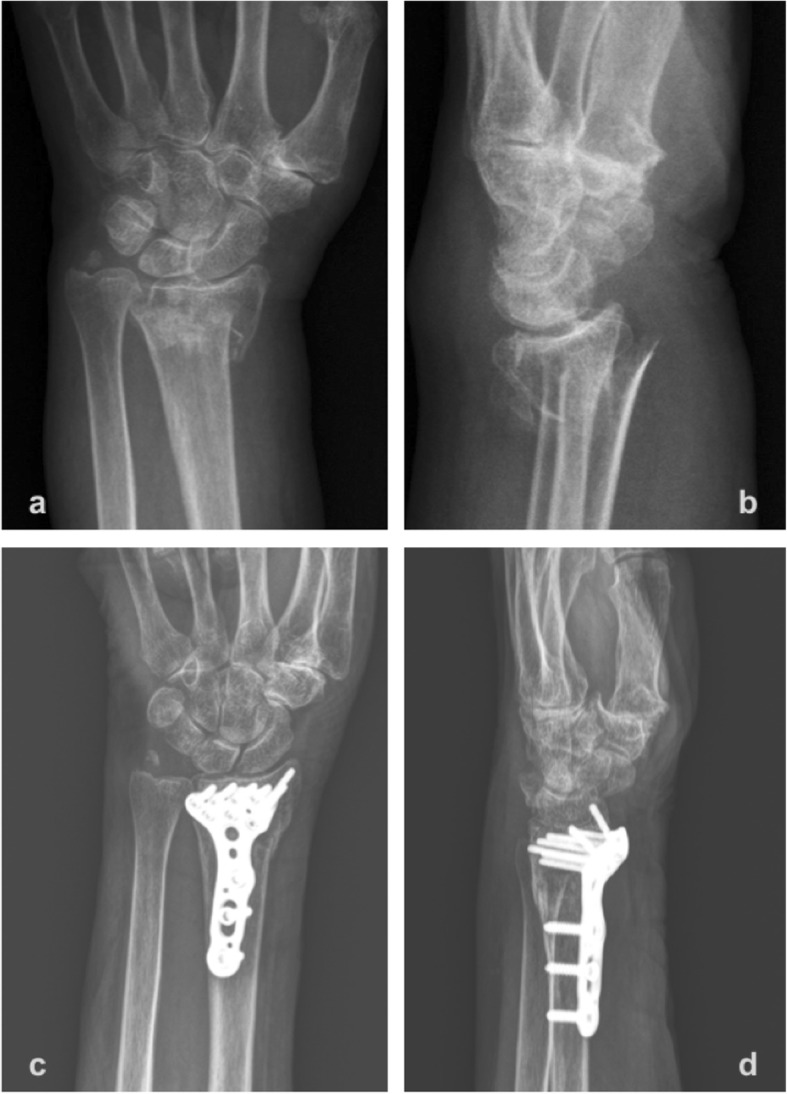
**a**, **b**; The anteroposterior and lateral plain films showed an intra-articular comminuted distal radius fracture. **c**, **d**; Immediate postoperative anteroposterior and lateral plain films presented after adequate reduction and volar plating fixation

**Fig. 2:**
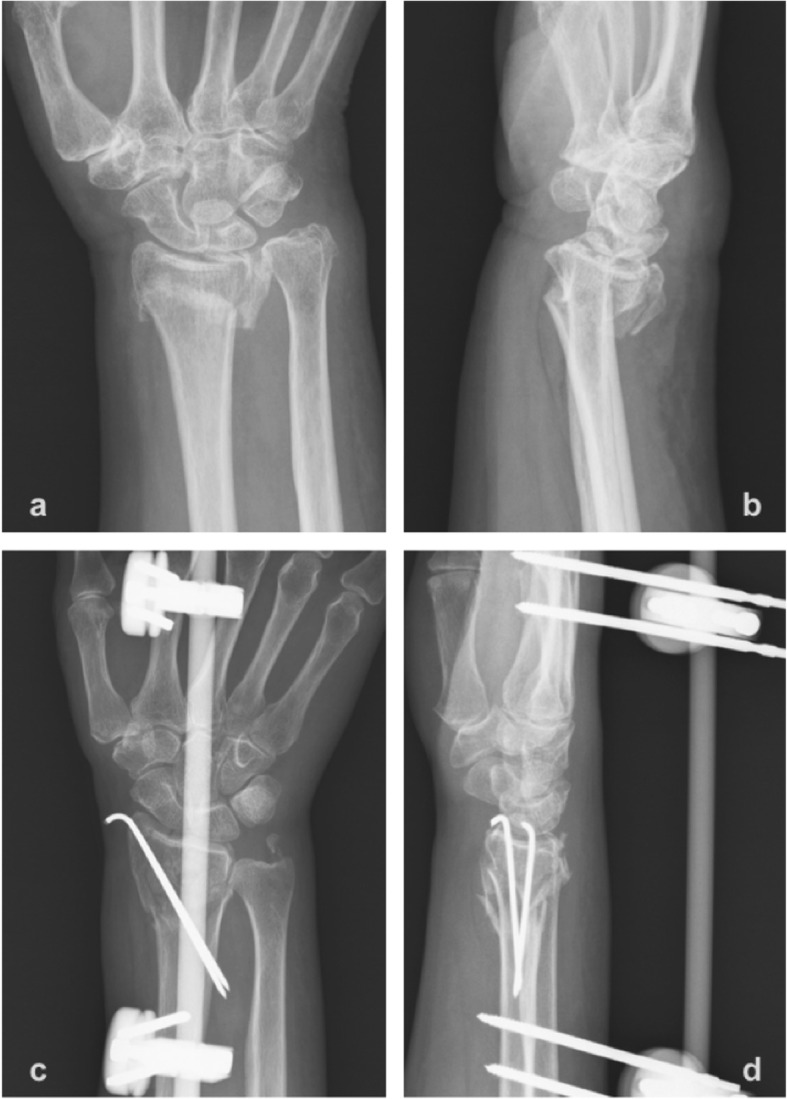
**a**, **b**; The anteroposterior and lateral plain films showed an intra-articular comminuted distal radius fracture with dorsal displacement. **c**, **d**; Immediate postoperative anteroposterior and lateral plain films presented after adequate reduction, external fixator and K-pins fixation

The patients were followed up at the outpatient clinic 2 weeks, 6 weeks, 3 months, 6 months, 1 year, and 2 years postoperatively. Postoperative immediate radiographs and anteroposterior- and lateral-view radiographs in the final follow-up clinic were evaluated (Fig. 1 and Fig. 2). Volar tilt, radial inclination, radial height, and ulnar variance were recorded and compared with the values on the preoperative plain radiographs [13]. The wrist range of motion was assessed in the final clinical visit. Flexion-extension, and forearm supination-pronation were measured by a goniometer. Any postoperative complications, including infection, neuropathy, complex regional pain syndrome, tendonitis, implant failure, malunion, or nonunion, were also evaluated retrospectively for every patient at each clinical visit.

### Statistics

Continuous data are shown as mean ± standard deviation. As the preliminary Kolmogorov-Smirnov test showed that the samples did not follow a normal distribution, we used the Mann-Whitney U test to compare the continuous variables between the external fixator and VLP group. Fisher’s exact test and the chi-squared test were used to compare the categorical variables between the two groups. Within these analyses, values of *p* < 0.05 were considered statistically significant.

## Results

### Demographic data

Forty-one (6 men, 35 women) with a mean age of 84 years (80–97) were treated with external fixators and 28 (4 men, 24 women) with a mean age of 84 (80–96) were treated with a VLP. 24 patients (24/41) in the external fixators and 19 (19/28) in the VLP group were intra-articular fracture. AO fracture types included A2 (*n* = 11), A3 (*n* = 8), C1 (*n* = 6), C2 (*n* = 6), and C3 (*n* = 10) in the external fixator group and A2 (*n* = 9), A3 (*n* = 2), C1 (*n* = 7), C2 (*n* = 5), and C3 (*n* = 5) in the VLP group. The operation time was shorter in the EF group (58 (SD19) vs 97 (SD29) mins, *p* < 0.01). The length of hospital stay was shorter (2 ± 1 days vs 3 ± 1 days) in the EF group. Average follow-up time was 1.3 years (1–1.9) in EF group and 1.4 years (1–1.9) in VLP group. The patients’ demographic data are shown in Table 1.

**Table 1:** Demographic data of the patients

	External fixation (*n* = 41)	Volar locking plate (*n* = 28)	*p* value
Mean age, years (range)	84 (80–97)	84 (80–96)	0.53
Male/Female	6/35	4/24	0.97
Type of fracture			0.46
Extra-articular	17 (41%)	9 (32%)	
Intra-articular	24 (59%)	19 (68%)	
OTA classification			0.51
A2	11 (27%)	9 (32%)	
A3	8 (19%)	2 (7%)	
C1	6 (15%)	7 (25%)	
C2	6 (15%)	5 (18%)	
C3	10 (24%)	5 (18%)	
Operation time, mins	58 ± 19	97 ± 29	**< 0.0001**
Hospital stay, days	2 ± 1	3 ± 1	**0.0002**
Follow-up time, years	1.3 ± 0.4	1.4 ± 0.4	0.38

Significant *p* values are shown in bold. *CVA* Cerebrovascular accident, *CAD* Coronary artery disease, *OTA* Orthopaedic Trauma Association

### Radiographic outcomes

The radiographic parameters of the preoperative, postoperative, and final plain radiographs are shown in Table 2. There were no significant differences in radial height, ulnar variance, radial inclination, and palmar tilt between the pre- and immediate postoperative groups. At the final follow-up radiographic parameters, there was no significant difference in the radial height and palmar tilt between EF group and VLP group. However, the ulnar variance was 3.4 ± 2.8 mm in the EF group and 1.8 ± 3.2 mm in the VLP group, a difference that was statistically significant (*p* = 0.01). Besides, the radial inclination was 19.1 ± 5.9 ° in the EF group and 22.1 ± 5.2 ° in the VLP group, which was also statistically significant (*p* = 0 .03).

**Table 2:** Comparison of radiographic data between EF group and VLP group

	External fixation (*n* = 41)	Volar locking plate (*n* = 28)	*p* value
Preoperative			
Radial height (mm)	5.4 ± 4.6	6.9 ± 3.9	0.19
Ulnar variance (mm)	5.1 ± 3.4	4.8 ± 4.1	0.59
Radial inclination (°)	12.8 ± 7.2	15.0 ± 7.5	0.23
Palmar tilt (°)	−10.6 ± 18.4	−12.6 ± 16.3	0.41
Associated ulnar styloid fracture	23	11	0.22
Postoperative day 1			
Radial height (mm)	10.1 ± 2.9	11.2 ± 2.9	0.21
Ulnar variance (mm)	1.2 ± 2.4	1.4 ± 2.3	0.68
Radial inclination (°)	21.7 ± 32.4	22.8 ± 5.0	0.63
Palmar tilt (°)	0.4 ± 8.5	2.8 ± 8.5	0.25
Final follow-up			
Radial height (mm)	8.8 ± 3.2	11.2 ± 5.4	0.06
Ulnar variance (mm)	3.5 ± 2.8	1.8 ± 3.2	**0.01**
Radial inclination (°)	19.1 ± 5.9	22.1 ± 5.2	**0.03**
Palmar tilt (°)	1.7 ± 9.9	−0.32 ± 8.0	0.37

Significant *p* values are shown in bold

### Wrist range of motion

The wrist range of motion in the last follow-up clinic is better in VLP group compared with EF group. In result, there is significant difference in forearm supination (74.7 ± 6.6 ° in EF vs 80 ± 7.2 ° in VLP, *p* = 0.002). Table 3 lists the data in detail.

**Table 3:** Comparison of wrist range of motion between EF group and VLP group

	External fixation (*n* = 41)	Volar locking plate (*n* = 28)	*p* value
Flexion (°)	61.9 ± 10.0	65.2 ± 7.6	0.15
Extension (°)	58.6 ± 7.7	61.1 ± 11.6	0.28
Supination (°)	74.7 ± 6.6	80 ± 7.2	**0.002**
Pronation (°)	78.5 ± 8.6	82.5 ± 8.0	0.054

Significant *p* values are shown in bold

### Complications

The overall postoperative complication in both groups are shown in Table 4. 22 patients in the EF group and five patients in the VLP group presented one or more complications. Significantly fewer complications were seen in the VLP group (*p* = 0.003). 11 patients in the EF group and one patient in the VLP group developed a pin tract or wound infection, and all required oral antibiotic treatment. 6 patients in the EF group and 2 in the VLP group had wrist stiffness and required longer physiotherapy durations. Five patients in the EF group and two in the VLP group had tendonitis that was resolved by pain killers and rehabilitation. One patient in the EF group and one patient in the VLP group experienced neuropathy; both recovered spontaneously. In the EF group, 2 patients suffered from chronic regional pain syndrome and were treated with analgesics. 2 patients experienced pin tract loosening and were treated with fixator removal and splinting (28 days and 53 days). No patients developed nonunion in the EF or VLP groups. There were no major perioperative complications related to the respiratory or gastrointestinal system.

**Table 4:** Complications

	External fixation (*n* = 41)	Volar locking plate (*n* = 28)	*p* value
Overall incidence of complications	22 (54%)	5 (18%)	**0.003**
Infection	11 (27%)	1 (4%)	**< 0.0001**
Chronic regional pain syndrome	2 (5%)	0 (0%)	0.24
Wrist stiffness	6 (15%)	2 (7%)	0.34
Tendonitis	5 (12%)	2 (7%)	0.50
Neuropathy	1 (2%)	1 (4%)	0.79
Implant failure	2 (5%)	0 (0%)	0.24

Significant *p* values are shown in bold

## Discussion

As medical care improves, elderly individuals desire to maintain their independence after low-energy trauma such as a distal radius fracture. Although many studies have evaluated the treatment outcomes of distal radius fractures in the elderly [14–20], no study has focused on the surgical treatments of distal radius fractures in patients aged > 80 years. In our study, final radiographic parameters showed a positive ulnar variance of 1.8 mm and a mean radial inclination of 22.1°. The wrist range of motion on the final clinic showed a mead supination of 80° which showed also a statistically better in the VLP group. These findings were supported by Schmelzer-Schmied et al. [19]. They studied 45 patients aged 50–70 years with C1/C2 distal radius fracture who underwent EF, plating with locking or non-locking volar plates and reported the significantly best radiological results and wrist range of motion of VLP compared with EF and non-locking plate methods. A randomized controlled trial by Williksen et al. [14] demonstrated less radial shortening and better supination after volar locking plating in 51 of 94 patients aged 20–84 years with unstable distal radius fractures at 52 weeks. Another randomized controlled trial by Navarro et al. [20] reported 140 patients aged 50–74 years with a displaced distal radius fracture who underwent ORIF with VLP or EF. The radiographic restoration of alignment was better for the VLP group than the EF group at the postoperative follow-up. And the wrist range of motion was equal in both groups except for the radial deviation, which was better in the VLP group at 1-year follow up by Navarro et al.

We observed significant differences in the overall incidence of complications between the groups. A higher incidence of infection was noted in the EF group, in which 11/41 (27%) patients treated with EF developed a pin tract infection that required oral antibiotic treatment versus 1/28 (4%) patients in the VLP group. The overall complication and infection rates of the EF group were higher in our study than in others [14, 19, 21, 22]. Navarro et al. [20] demonstrated that the total complication rate was equal between the EF and VLP groups. In a meta-analysis, Yuan et al. [23] demonstrated that EF yielded a higher incidence of total complications, infection, and malunion in patients aged ≥16 years. Our study was supported by these findings. Perhaps a younger skeletally mature population would demonstrate fewer complications for EF than in our study. We consider our findings valuable for patients aged > 80 years.

As the incidence of osteoporosis is higher in elderly populations, the metaphysis of the bone is weaker after reduction, resulting in large metaphyseal voids that caused fracture instability [24]. Our study showed that the VLP group met the more acceptable radiological parameters at union, better wrist range of motion at average 1-year follow-up and lower complication rates in patients aged > 80 years. We believe that the VLP design was suitable for maintaining the reduction and increasing the stability in elderly patients with osteoporosis or comminuted fracture [25]. Although EF had the advantages of easy application, lower cost than VLP, it carried higher risks of poor postoperative radiographic parameters and pin tract loosening, especially in osteoporotic bone.

There were limitations to our study. First, this retrospective study does suffer from selection bias. Second, the choice of surgical method depended on discussion between the patient/family and surgeons. The increased cost of VLP may be a concern in the medical decision-making process. Third, we were unable to collect other outcome questionnaires due to the retrospective nature of this study.

The main strength of this study included that we evaluated the effect of surgical treatment on distal radius fracture in patients aged > 80 years, as previous studies assessed patients aged ≥65 years. Second, it included a large number of patients in both groups with no important demographic differences. We think it is more important to assess the fracture treatment outcomes in patients aged ≥80 years and hope that further prospective studies can validate our findings.

## Conclusion

VLP provides better radiological outcomes, wrist supination and lower complication rates than EF. Therefore, although EF is still widely used because of its acceptable results and easy application, we recommend VLP as a suitable treatment option for distal radius fracture in the geriatric population aged > 80 years.

## Data Availability

The data which analyzed during the study are stored in our hospital and are available from the corresponding author on reasonable request.

## Abbreviations

EF: External fixation
ORIF: Open reduction internal fixation
VLP: Volar locking plate
